# The biological function of Serpinb9 and Serpinb9-based therapy

**DOI:** 10.3389/fimmu.2024.1422113

**Published:** 2024-06-20

**Authors:** Haozhe Huang, Yiqing Mu, Song Li

**Affiliations:** ^1^ Department of Pharmaceutical Sciences, University of Pittsburgh School of Pharmacy, Pittsburgh, PA, United States; ^2^ University of Pittsburgh Medical Center (UPMC) Hillman Cancer Center, University of Pittsburgh, Pittsburgh, PA, United States

**Keywords:** SerpinB9, granzyme B, immunotherapy, immune cell, nanotechnology

## Abstract

Recent breakthroughs in discovering novel immune signaling pathways have revolutionized different disease treatments. SERPINB9 (Sb9), also known as Proteinase Inhibitor 9 (PI-9), is a well-known endogenous inhibitor of Granzyme B (GzmB). GzmB is a potent cytotoxic molecule secreted by cytotoxic T lymphocytes and natural killer cells, which plays a crucial role in inducing apoptosis in target cells during immune responses. Sb9 acts as a protective mechanism against the potentially harmful effects of GzmB within the cells of the immune system itself. On the other hand, overexpression of Sb9 is an important mechanism of immune evasion in diseases like cancers and viral infections. The intricate functions of Sb9 in different cell types represent a fine-tuned regulatory mechanism for preventing immunopathology, protection against autoimmune diseases, and the regulation of cell death, all of which are essential for maintaining health and responding effectively to disease challenges. Dysregulation of the Sb9 will disrupt human normal physiological condition, potentially leading to a range of diseases, including cancers, inflammatory conditions, viral infections or other pathological disorders. Deepening our understanding of the role of Sb9 will aid in the discovery of innovative and effective treatments for various medical conditions. Therefore, the objective of this review is to consolidate current knowledge regarding the biological role of Sb9. It aims to offer insights into its discovery, structure, functions, distribution, its association with various diseases, and the potential of nanoparticle-based therapies targeting Sb9.

## 1 Overview of Serpinb9

Serpinb9 (Sb9), first defined in 1995 (1), is a member of the largest and most widely distributed superfamily of proteinase inhibitors known as serpins, which specifically inhibit serine proteases (2). The serpins superfamily is sub-divided into 16 clades (A-P) based on their phylogenetic relationships (2). These proteins are designated SERPINXy, where ‘X’ signifies the specific clade and ‘y’ denotes the individual number within that clade (2). To date, there have been no less than 37 serpins identified in humans (3). Among these, the extracellular ‘clade A’ serpins and the intracellular ‘clade B’ serpins represent the two largest categories within the human serpin family (4). While the majority of serpins primarily inhibit serine proteases, it’s worth noting that there have been identified cross-class inhibitors that target caspases (5, 6) and papain-like cysteine proteases (7, 8) as well.

Serpins exert their inhibitory effects on proteases, contributing to various physiological processes, including inflammation, complement activation, blood coagulation, tissue remodeling and immune response as well as prevention of inappropriate activity of cytotoxic proteases induced apoptosis (9). Dysfunction within the serpin family is implicated in a wide range of pathophysiological processes and diseases. Several conformational diseases or ‘serpinopathies’ linked to polymerization of serpins within the endoplasmic reticulum have been identified (10). These include emphysema (SERPINA1 mutation) (11), thrombosis (SERPINC1 deficiency) (12), hereditary angioedema (SERPING1 deficiency) (13), cirrhosis (SERPINA1 polymerization) (14) and Alzheimer’s disease (SERPINA3 polymorphism) (15).

All serpin proteins share a conserved tertiary structure characterized by a core domain with three β−sheets, eight-nine α−helices, and a flexible reactive center loop (RCL) that contains seventeen amino acids and is positioned in the protease recognition domain close to the C−terminus of serpin (16). The core structure of serpins is highly conserved, which determines the specificity of serpin inhibitory functions (17). Sb9 is a single-chain protein composed of 376 amino acids with a molecular weight of 42 kDa (18). Consistent with other members in the serpin superfamily, Sb9 possesses a highly preserved tertiary structure. It inhibits GzmB by initially forming a reversible Michaelis complex, followed by the formation of a covalent complex: a covalent ester linkage is formed between the sidechain oxygen atom of GzmB and the carbonyl carbon of Sb9 P1 residue (19–21). In addition, Losasso et al. find that R28K, R201A, and R201K GzmB mutants substantially disrupt the interaction with Sb9. In particular, the activity of R201K GzmB closely resembles that of the wild-type protein, regardless of the presence or absence of Sb9 (22). It is important to note, however, that the structure of human Sb9 has not been fully elucidated. Consequently, additional research is necessary to validate these findings.

Sb9 is extensively expressed in both the cytoplasm and nuclei of various human endothelial, mesothelial and immune cells, particularly in immune-privileged sites such as placenta, testes, ovaries and eyes (23–25). Furthermore, Sb9 is present at high and relatively stable levels in cytotoxic lymphocytes (comprising natural killer cells and CD8^+^ T cells), as well as in antigen-presenting cells (24, 26).

As previously noted, Sb9 is a potent physiological inhibitor of GzmB through formation of protein complex. GzmB, a caspase-like serine protease, triggers apoptosis of virus-infected cells or tumor cells by activating caspases-3 and -8 (27). Sb9 can keep the GzmB staying in the cytoplasm (28). Subcellular fractionation experiments show that Sb9 can be imported and exported through nuclei to grab any GzmB that evades the nucleocytoplasmic pool of Sb9 (23).

It has been established that Sb9 can protect tumor cells from GzmB mediated destruction, whether GzmB originates from cytotoxic lymphocytes or is produced endogenously (29, 30). Moreover, Sb9 has been revealed to provide cytoprotective effects against self-inflicted injury caused by GzmB in pro-inflammatory leukocytes (cytotoxic T cells (31), dendritic cells and neutrophils) or anti-inflammatory leukocytes (regulatory T cells [T_regs_] and myeloid-derived suppressor cells [MDSCs]) (30–33).

It’s worth noting that apart from its role in regulating GzmB, human Sb9 can also influence cytokine production by modulating inflammatory caspase-1, and to a lesser extent caspase-4 and caspase-8, but not caspase-3 (34). Sb9 accounts for the innate inhibitory activity against caspase-1 through protein-protein interaction and prevents processing of the natural substrates of caspase-1, specifically IL-1β and IL-18 precursor in both human vascular smooth muscle cells and monocytes (35, 36). Additionally, there are reports suggesting that Sb9, functioning as a protease inhibitor, impedes tumor necrosis factor- and Fas-induced apoptosis by directly interacting with the pro-apoptotic apical caspases-8 and -10 (37, 38). However, considering the low K_asso_ (association rate constant) value and lack of data on Sb9 RCL cleavage or caspase-Sb9 complex formation, the physiologically significant interaction between Sb9 and these caspases is limited and unpersuasive (39, 40).

Besides as a protease inhibitor, several studies have indicated murine Sb9 functions in the regulation of antigen presentation and the cell-associated antigen cross-presentation by CD8^+^ splenic dendritic cells, independently of GzmB (41, 42). In addition, Sb9 has been described to inhibit the activity of subtilisin A and neutrophil elastase rather than human furin via a rapid and single step mechanism (43, 44).

Considering the vital role of Sb9 in numerous physiological functions, gaining a deeper insight into its regulation, encompassing both intracellular factors and extracellular environment, could offer profound implications for clinical practice. Delving into these regulatory mechanisms might pave the way for innovative therapeutic interventions, diagnostics, or preventive measures, especially in those conditions where dysregulation of Sb9 is a key factor. Estrogen is first shown to boost the expression of Sb9 significantly and rapidly in an estrogen receptor-positive human liver cell line. This induction occurs through the Sb9 promoter region by the transcription factor estrogen receptor α (ERα) (45–47). The presence of EGF and the activation of ERK further enhance the estrogen-driven induction of Sb9 (46, 47). These results indicate estrogens regulate the susceptibility of target cells to immune system-induced apoptosis. Another two factors, IL-1β and IL-18, while being downstream targets of Sb9, are also pinpointed as inducers of Sb9 via the transcription factors nuclear factor-κB (NF-κB) and activator protein-1 (AP-1) (48, 49). Notably, mutational ablation of NF-κB and AP-1 binding sites almost entirely suppresses the IL-1β-induced expression of Sb9 (48). Recent studies have uncovered that Sb9 can be modulated by MUC1-C-IFNγ oncogenic signaling via the interferon regulator factor 1 (IRF1) transcription factor and by type 1 interferon in murine tumor cells (50–52). Additionally, cytokines are involved in regulating Sb9 expression in liver cells. Exposure of murine hepatocytes to IFN-α/β/γ induces Sb9. In human hepatoma Huh-7 cells, treatment with IFN-α/γ or TNF-α elevates Sb9 expression, while the related serine proteinase inhibitor, serpinB8, remains unaffected (53). Moreover, the IFN-α-induced upregulation of Sb9 in hepatocytes is specifically triggered by the infiltration of NK cells in the liver, which carry active GzmB and perforin (54). Furthermore, high Sb9 content in breast stem tumorspheres and dendritic cells, expressing high levels of CXCR4 and phospho-p38, might be ascribed to the activation of the CXCR4/p38MAPK proliferative signaling pathway (55, 56). Other factors, including STAT1 and NME1, have been reported to positively correlate with Sb9 expression (57, 58). Granzyme M acts as an endogenous inhibitor of Sb9, with human granzyme M potentially clearing the way for GzmB-mediated target cell damage by inactivating Sb9, a mechanism by which natural killer cells might overcome this escape mechanism (59).

Beyond the endogenous regulators, exogenous compounds have also been shown to affect the expression of Sb9. For example, lipopolysaccharide and 12-O-tetradecanoylphorbol-13-acetate have been shown to induce its expression in HepG2 cell line (48). Pentoxifylline stimulates the expression of Sb9 in K562 cells at the mRNA level, but not protein level (60). Additionally, low concentrations of the soy phytoestrogen genistein have been found to induce Sb9 and subsequently hinder killing of ERα^+^ breast cancer cells by immune cells (61). This suggests that soy consumption levels may be a relevant consideration for ER^+^ breast cancer patients (61). Cigarette side stream smoke particulate matter (CSSP), a major component of secondhand smoke, upregulates Sb9 in a dose-dependent manner through the activation of ERα in human lung adenocarcinoma cells (62). In glioma cell lines, Sb9 is also elevated by low pH 6.5, threefold or more, in both 2D tumor cells and 3D glioma stem cells. These findings imply that culturing cancer cells in lower pH conditions, which mimic the endogenous tumor environment, leads to a marked rise in Sb9 and more accurately preserve tumor heterogeneity (63). The upregulation of Sb9 can be mitigated by reactive oxygen species, which inactivate Sb9 by oxidizing a highly conserved cysteine pair in the reactive center loop to form a vicinal disulfide bond (64). Interestingly, we have recently discovered that the expression of Sb9 is significantly induced in both gemcitabine resistant pancreatic cancer cell lines and following transient exposure to gemcitabine. Mechanistic study shows that gemcitabine induces Sb9 expression at transcriptional level through transcription factor ATF3 (unpublished data). Importantly, Sb9 stable knockout (KO) or transient KD via siRNA leads to increased sensitivity to gemcitabine treatment, providing the 1^st^ evidence that Sb9 is also involved in regulation of drug sensitivity in cancer cells (unpublished data).

## 2 Distribution and function of Serpinb9 in different cell types

The primary function of Sb9 is to safeguard somatic cells such as stem cells, immune cells and tumor cells from apoptosis through diverse pathways (65–70). Sb9 plays a dual role in immunity, especially in the regulation of different immune cells and tumor cells. Within immune cells such as cytotoxic T lymphocytes, the primary role of Sb9 is to protect them from apoptosis and cell death mediated by the self-produced GzmB. It ensures that immune cells can utilize GzmB to eliminate target cells without falling prey to their own cytolytic mechanisms. Comprehending the distinct role of Sb9 in modulating various signaling pathways within immune cells is essential for enhancing immune function. However, when co-opted by tumor cells, Sb9 can facilitate immune escape (71), highlighting its intricate role within the immune landscape. Its overexpression in certain cancers and cancer stem cells might be associated with tumor initiation and metastasis, therapy resistance, and epithelial-to-mesenchymal transition (EMT).

**Figure 1:**
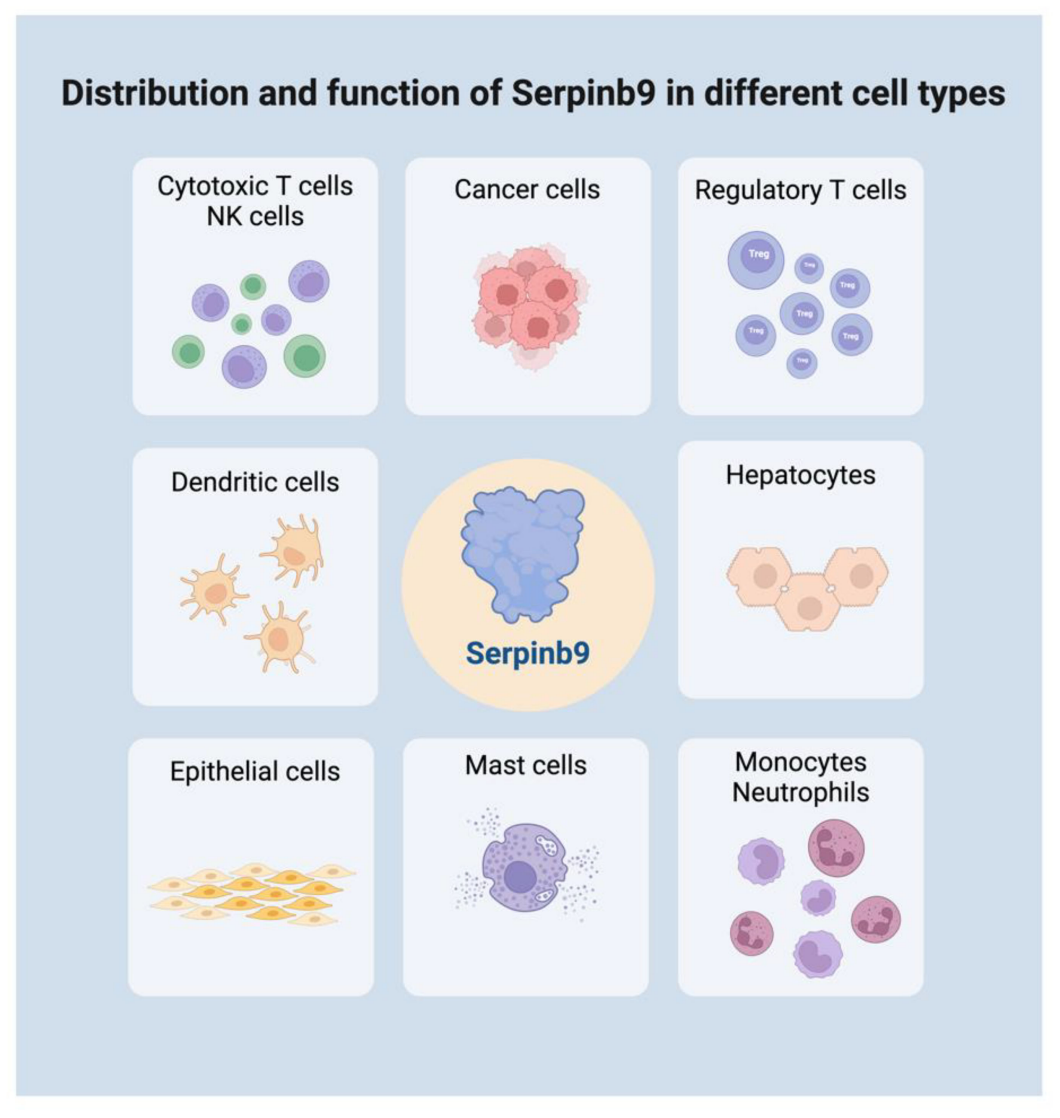
Distribution and function of Serpinb9 in different cell types. Serpinb9 is expressed in various immune cells, somatic cells, and tumor cells. It plays a dual role in immune modulation across different cell types. Created with BioRender.com.

Although Sb9 is primarily recognized for its involvement in immunity and cancer, there is evidence Sb9 is involved in other physiological and pathological processes as well. Understanding the differential expression of Sb9 across cell types and tissues is vital for deciphering its physiological and pathological roles in, for example, hepatitis, renal allograft, and autoimmune disorders. It is important to note that while we have general knowledge about Sb9’s roles and expression profiles, the exact levels and functional significance might vary based on the specific tissue or disease context. This knowledge can help devise therapeutic strategies, especially in diseases like cancer where Sb9 expression might influence disease progression and treatment outcomes (**Figure 1**).

### 2.1 Cytotoxic T cells and NK cells

Sb9 emerges as a signature gene of T cells dysfunction from the “statistical interaction test”, which is employed to identify specific genes in several cancer types that could be linked to patients’ abnormal tumor immune microenvironment. The identification of Sb9 as a hallmark of T cell dysfunction implies that the presence or high expression of this gene in a tumor immune microenvironment can serve as a biomarker of tumor immune evasion (50, 72).

It is well established that the appearance of GzmB in the cytoplasm of NK cells and cytotoxic T lymphocytes (CTLs) during activation is responsible for cell death. Restimulation of previously activated T lymphocytes induces lysosomal membrane permeabilization, followed by GzmB release into the cytosol (73). Sb9 acts to neutralize misdirected GzmB following the degranulation of cytotoxic cells. As a result, the suppression of GzmB by Sb9 can enhance the viability of CTLs and NK cells (73–75). This mechanism potentially explains why cytotoxic cells are not damaged by their own GzmB during destruction of abnormal cells (76). The Sb9 is also upregulated during degranulation of effector cells, and its overexpression enhances CTLs potency by improving cell viability (26). Research indicates that the absence of the Sb9 gene renders CTLs more vulnerable to premature death and diminishes their effectiveness in recognizing and eradicating virus-infected cells (31). Invariant natural killer T (iNKT) cells from Sb9 knockout mice exhibit impaired production of IL-4 and IFN-γ, highlighting that Sb9 is required for immunoregulatory function of iNKT cells (77). Similarly, Sb9 plays a critical role in protecting NK cells from GzmB-mediated cell death. Sb9^-/-^ mice are highly susceptible to *Ectromelia* virus infection, which is attributed to NK cells being more prone to GzmB-mediated death, leading to diminished cytotoxic potential (75, 78).

### 2.2 Effector T cells and regulatory T cells

Sb9 exhibits varied functions across different T cell subtypes from diverse origins. For example, CD8^+^ effector T cells are categorized into those destined to become long-lived memory CD8^+^ T cells (memory-precursor-effector-CD8^+^-cells [MPEC]) and those that are short-lived (short-lived-effector-CD8^+^-cells [SLEC]). The formation of SLEC is affected by Sb9, but not MPEC, suggesting that Sb9 has a differential impact on the survival of effector and memory alloreactive CD8^+^ T cells (79). Generally, overexpression of Sb9 in mice leads to an increased frequency and enhanced development of memory T cells (80). Additionally, the expression of Sb9 serves to ensure the survival of metabolically active CD8^+^ memory T cells, protecting them from the effects from cytotoxic T lymphocytes (80). However, if memory CD8^+^ T cells originate from the progenitors that are refractory to self-inflicted damage with low levels of Sb9 and GzmB, rather than from fully differentiated CTLs, Sb9 is not required for the development and survival of these subsets of memory CD8^+^ T cells (81). Intriguingly, TCRαβ^+^CD3^+^CD4^-^CD8^-^NK1.1^-^ double-negative regulatory T cells have been observed to suppress CD8^+^ memory T cell but not CD4^+^ memory T cells, which is attributed to the high expression of Sb9 in CD4^+^ memory T cells (82).

Regulatory T cells (T_regs_) are critical for maintaining immune homeostasis by suppressing the activity of other immune cells, thus preventing autoimmunity and excessive inflammation. Alterations in function or quantity of T_regs_ can result in immune dysregulation. Interestingly, Sb9 has been found to have a contentious role in T_regs_ across various diseases, a topic that continues to be at the forefront of intense research and debate. In murine skin graft model, GzmB^+^ T_regs_ exhibit an immunosuppressive role, contributing to prolonged graft survival. Overexpression of Sb9 interferes with the function of GzmB in T_regs_ and results in decreased graft survival compared to tolerized wild-type (WT) mice (83). In contrast, another research shows that Sb9 is critical for maintaining the potency and of survival of activated T_regs_ in graft versus-host disease, where it exerts immunosuppressive functions (32). Prashant S. Giri et al. identify that T_regs_ from generalized vitiligo patients express lower level of Sb9 compared to control. The decreased Sb9 lead to the reduced number of T_regs_, implicating a role in the pathogenesis of generalized vitiligo (84). In the context of HIV, some infected CD4^+^ Th1 cells express Sb9, which shields these infected cells from their own GzmB mediated and CD8^+^ T cells mediated killing. These observations hint at a potential mechanism for HIV-1 RNA^+^ cells to evade destruction by cytotoxic CD8^+^ T cells: Sb9 could reside within cytotoxic CD4^+^ T cells that are inherently resistant to GzmB mediated death. However, further studies involving more extensive single-cell evaluations are essential, given the limited scope of cells analyzed in these studies (85).

### 2.3 Dendritic cells

Dendritic cells display elevated levels of Sb9 in primary lymphoid organs and inflammatory sites. This presence is noted in thymic DCs (characterized as CD3^-^, CD4^+^, CD8^-^, CD45^+^), tonsillar DCs, DC subsets from peripheral blood (CD16^+^ monocytes and CD123^+^ plasmacytoid DCs), as well as monocyte-derived DCs (24, 26). Furthermore, Sb9 is tightly associated with the maturation of DCs (increased levels of DCs activation marker CD86) induced by TNF-α, LPS, CD40L or ectromelia virus infection (26, 42, 55, 86). The elevated expression of Sb9 is found to be functionally significant for protecting DCs, as LPS-treated DCs become less vulnerable to CTL-mediated killing *in vitro* (86). Further evidence shows that impaired survival of CD8α DCs is observed in Sb9 knockout mice following infection with lymphocytic choriomeningitis virus (55, 87). However, when these LPS-treated DCs are injected *in vivo*, they remain susceptible to CTL-mediated killing. This is observed irrespective of whether the CTLs in host mice are elicited via active immunization or passively transferred via the injection of *in vitro*-activated CTLs. A rapid reduction in Sb9 expression levels in DCs after *in vivo* injection may explain why DCs are resistant to CTL-mediated killing *in vitro* but not *in vivo* (86).

In addition, Sb9 acts as a biomarker for antigen cross-presenting DCs, CD8^+^DC subsets. Notably, only the subset with high Sb9 expression is capable of cross-presentation (42). On the contrary, DCs from Sb9-deficient mice demonstrate a compromised ability to cross-present cell-associated antigens and cross-prime CTLs, with this deficit being independent of GzmB (41).

Furthermore, a novel subpopulation of IFN-induced DCs with high levels of Sb9 is identified. These cells exhibit direct tumoricidal activity against K562 cells, deserving further study to become a potential regimen for cancer therapy (88).

### 2.4 Mast cells

The recent discovery of Sb9 expression in mast cells, known for their central role in allergic responses and innate immunity, adds to its established presence in T cells and DCs. This finding highlights Sb9’s potentially extensive impact on immune regulation. Activation of mast cell-lines HMC-1 and LAD2, as well as cord blood- and mature skin-derived human mast cells by PMA and calcium ionophore A2318 results in expression and release of GzmB, accompanied by an upsurge in Sb9 expression (89, 90). Similar to cytotoxic lymphocytes, Sb9 is localized in the cytoplasm of mast cells. Furthermore, GzmB has been observed in granules within mast cells that are morphologically akin to the cytotoxic granules seen in CLs, as revealed through immunohistochemical staining and immuno-electron microscopy. This upregulation of Sb9 in activated mast cells strongly suggests that Sb9 serves to protect these cells against apoptosis induced by either autologous misdirected GzmB or GzmB released during the onset of local inflammatory responses, mirroring observations in CLs (89, 90). While the direct link between Sb9 and mast cell activation is yet to be comprehensively elucidated, it is conceivable that Sb9’s modulation of GzmB activity could influence the release of mediators from mast cells, thereby impacting allergic reactions, asthma and inflammatory responses. Incorporating the role of Sb9 in mast cells into diagnostic or therapeutic strategies can provide a holistic approach, especially in conditions influenced by mast cell activity.

### 2.5 Monocytes

Upregulation of Sb9 is observed in lymphocytes and monocytes of patients with acute Epstein-Barr virus infection. Moreover, Sb9 is upregulated by IL-2 and LPS in monocytes, but not lymphocytes. This upregulation can be inhibited by NF-κB inhibitor pyrrolidine dithiocarbamate and dexamethasone, indicating a relevant biological role of Sb9 in the functionality of leucocyte subsets during inflammatory processes (91).

During another inflammatory response triggered by *M. tuberculosis* infection, a rapid increase in Sb9 levels is observed in both alveolar macrophages and blood monocytes, lasting over seven days. The induction of Sb9 not only disrupts the cell death process but also promotes the survival of both host cells and the intracellular pathogen. These findings suggest that Sb9 might have a multifaceted impact on the progression of human *M. tuberculosis* infection, due to its roles in reducing inflammation and protecting against GzmB-triggered cell damage (92).

### 2.6 Neutrophils

Another function of Sb9 is an intracellular inhibitor of neutrophil elastase, a neutral serine protease released by polymorphonuclear neutrophils during inflammation and in response to Pseudomonas aeruginosa infection (44). Sb9 can also protect neutrophils from self-damage. Ablation of Sb9 in neutrophils can increase the neutrophil elastase activity in the extracellular environment due to elevated neutrophil lysis, therefore boosting neutrophil immunity against Pseudomonas aeruginosa infection (93). Additionally, upregulation of Sb9 is positively correlated with infiltration of neutrophil and negatively correlated with infiltration of CD8^+^ T cell within tumor microenvironment of uveal melanoma patients, indicating the potential prognostic ability of neutrophils in this context (94).

### 2.7 Tumor cells

In 2002, Sb9 was identified for the first time as being expressed *in vivo* by human tumors, non-Hodgkin and Hodgkin lymphoma (95). This discovery leads to the proposal of a novel mechanism by which Sb9 may enable tumor cells to circumvent immune surveillance (95, 96). However, a subsequent study suggests that primary lymphomas or lymphoma cell lines, despite expressing Sb9, remain sensitive to perforin-dependent cytolysis. This sensitivity is later found to be attributed to the cytolysis caused by the perforin-dependent pathways, rather than the Sb9-targeted protein, GzmB (97). Recent studies have increasingly shown that Sb9 plays a significant role in cancer progression, contributing to increased aggression and treatment failures. For example, there is a robust negative correlation between constitutive Sb9 expression levels and the activity of GzmB in leukemia cells, indicating Sb9 can inhibit the cell apoptosis induced by GzmB (98). Moreover, human cancer cell lines stably expressing Sb9 demonstrate a heightened ability to resist apoptosis triggered by both granzyme A or GzmB and Fas/Fas ligand pathway (37). At the cellular level, overexpression of Sb9 via transfection in leukemia cell line K562 (99) and mastocytoma cell line P815 (100) results in resistance against NK cell-induced apoptosis. Furthermore, Sb9 has been detected in various cancer cell types, including HepG2 hepatocellular carcinoma cells (46, 47), MCF7 breast cancer cells (46, 47), LCLC-103 H and A549 lung cancer cells (101), as well as prostate cancer cells (102), modulating their apoptosis induced by the immune system. Interestingly, in a study involving 8 Ewing sarcoma cell lines, tumor cells remain susceptible to perforin/granzyme-induced apoptosis even in the presence of Sb9, possibly due to alternative pathways (103). Moreover, *in vivo* studies show that genetic ablation of Sb9 using CRISPR/Cas9 significantly impede tumor growth in various cancer models, including B16 (murine melanoma), 4T1 (murine breast cancer), LLC1 (murine lung cancer), and A375 (human melanoma) (30).

Mechanistic study shows that Sb9 in tumor cells affects NK cell-mediated killing by decelerating the detachment of NK cells from tumor cells, a process recognized as a marker of successful target cell elimination (99). The rate of NK cell detachment from target cell defines the potency of NK cell-mediated killing. Delayed detachment often results from either reduced NK cell cytotoxicity or increased resistance of target cells. Cytokine-stimulated activated NK cells detach earlier than resting NK cells, correlating with their potent cytotoxicity. Overexpression of Sb9 and surface CD107a in K562 decelerates NK cells detachment from target cells, consequently diminishing the effectiveness of NK cell-mediated killing (99).

Recently, the influence of Sb9 on immune cells within the tumor microenvironment has gained attention. Sb9 can be regarded as a tumor antigen, recognized by human leucocytes antigen (HLA) class I-restricted and tumor-reactive CTLs. Sb9-recognizing CTLs can produce IFN-γ, potentially playing a role in the anti-tumor immune response in cancer patients (104). However, in B16-Sb9 KO tumor, the levels of infiltrated T_regs_, TAMs, and MDSCs have no difference compared to B16-WT tumor, indicating Sb9 from tumor cells has minimal impact on the proportion of immunosuppressive cell populations in the TME (30). Interestingly, in the Sb9 KO tumors in Sb9 KO mice, there are increased ratios of CD8^+^, CD44^+^CD62L^-^CD8^+^, TNFα^+^CD8^+^, GzmB^+^CD8^+^, and IFNγ^+^CD8^+^, as compared with those from the WT tumor in WT mice (30). Additionally, the numbers of TAMs, MDSCs and CAFs are significantly lower in TME of Sb9 KO tumors in Sb9 KO mice than in the WT tumors in WT mice. The loss of Sb9 in T_regs_, TAMs, and MDSCs drive them more sensitive to GzmB-mediated killing within the TME, ultimately leading to less infiltration of these immunosuppressive cells into the tumor (30). Furthermore, co-administration of siSb9, GzmB and anti-PD1 significantly induces the ratio of mature DCs, CD4^+^ and CD8^+^ T cells, as opposed to a pronounced decrease in T_regs_ in the tumor tissue of murine B16 model, which implies an activated immune system (105).

It should be highlighted that Sb9 is correlated with metastasis formation as well as the generation and maintenance of cancer stem cells. This association is underscored by the observed correlation between Sb9 expression and the epithelial-to-mesenchymal transition, both *in vitro* and in cancer patients (101). Metastatic melanoma cells have exhibited overexpression of Sb9 (106). Compared to B16-WT tumor, B16-Sb9 KO tumor displays a reduction in metastatic melanoma cells within tumor-draining lymph nodes (TDLNs) (30). Intriguingly, while Sb9 overexpression is evident in metastatic cells, its knockdown results in only a marginal increase in the vulnerability of these cells to CTL-mediated destruction, suggesting that Sb9 expression might not be associated with the resistance of metastatic cells (106). Regarding cancer stem cells, estrogen is recognized for its potential to act as a stimulus in upregulating the Sb9 expression (56). However, in the MCF7 breast cancer tumorspheres, an elevated level of Sb9 was observed despite reduced levels of the ERα66. Treatment with estrogens increases Sb9 level while decreasing ERα66 isoform and promoting cell proliferation, as demonstrated by the increased expression of proliferating cell nuclear antigen as well as increased number and size of tumorspheres (56). Additionally, low pH stress promotes malignancy through the induction of stemness of glioma cells along with increased Sb9 levels (63). This underscores the complex role of Sb9 in cancer progression and its potential as a target for therapeutic intervention.

### 2.8 Hepatocytes

The expression of Sb9 in hepatocytes is not solely induced by IFN or TNF (53). Other factors, such as the presence of GzmB and perforin in NK cells and CTLs, can also trigger Sb9 expression in hepatocytes (54). However, SPI-8 or other Sb9 homologs (Serpinb9b/9c/9d/9e/9g) in hepatocytes could not be induced significantly following IFN-α stimulation or during viral infection (54). Notably, this increase of Sb9 is absent in mice depleted of NK cells or lacking perforin or GzmB (54). This result indicates a feedback mechanism in hepatocytes where the upregulation of Sb9 during viral infection is correlated with the influx of NK cells and CTLs, and exposure to GzmB and perforin (54). This upregulation of Sb9 protects hepatocytes from NK cells and CTLs-mediated apoptosis. Conversely, inhibition of Sb9 results in increased hepatocellular injury during viral infections, indicating heightened hepatotoxicity (107).

## 3 Serpinb9 in diseases

**Figure 2:**
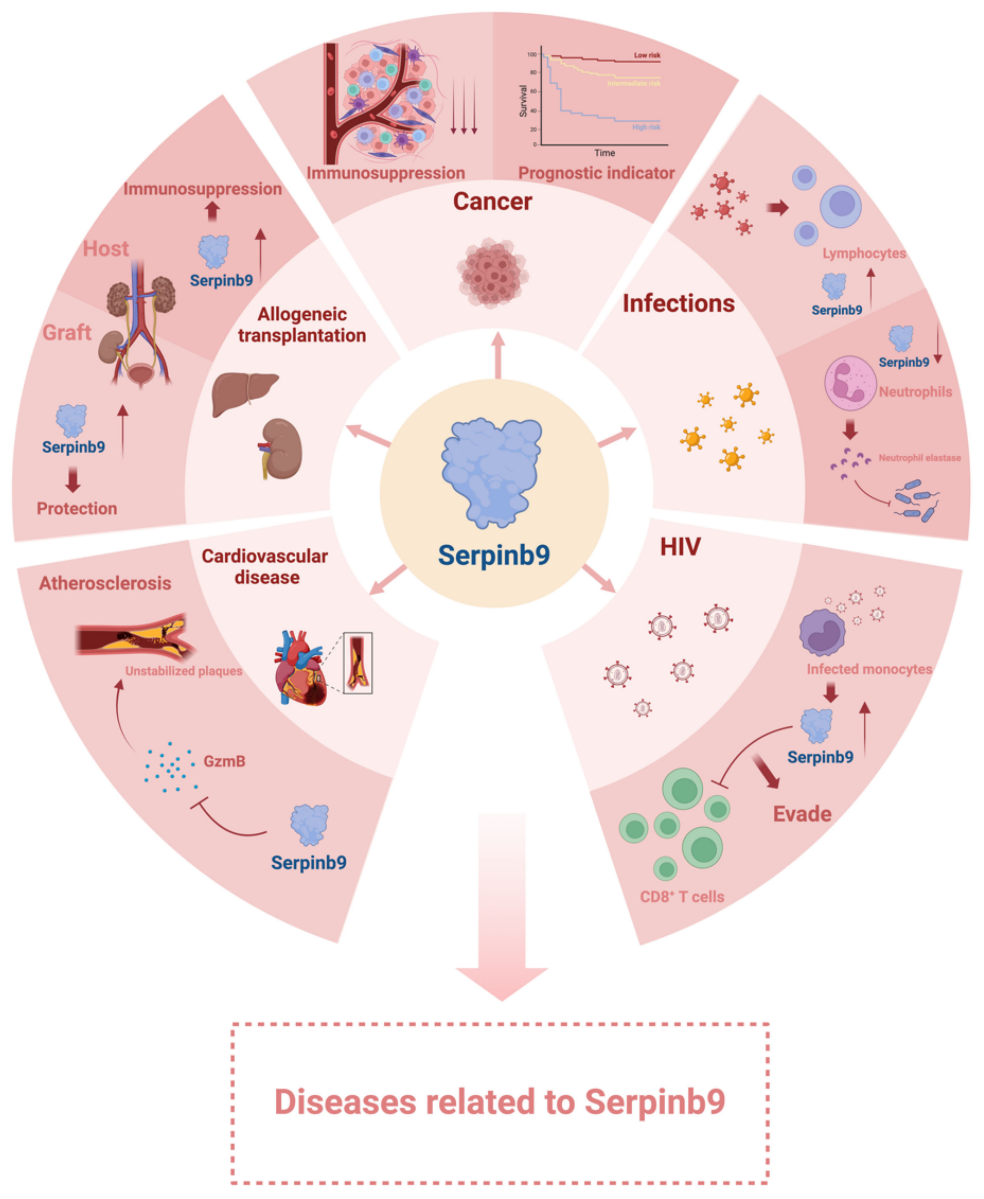
Diseases related to Serpinb9. Serpinb9 is involved in many biological processes. Disruptions in Serpinb9 function may cause various diseases. Created with BioRender.com.

Sb9 is involved in multiple biological pathways and plays a crucial role in regulating the cytotoxic activity of immune cells. Consequently, disruptions in Sb9 can lead to various immune and inflammatory diseases. For example, Sb9 A329S variant contributes to the pathogenesis of autoinflammatory disease due to its impaired ability to inhibit caspase-1 and IL-1β, indicating Sb9 might play an important role in autoimmune diseases (36). Moreover, impaired balance between Sb9 and GzmB is associated with pathophysiological processes of a spectrum of other health issues, including hepatitis (53, 54, 107), pancreatitis (108), non-alcoholic steatohepatitis (109), severe generalized vitiligo (84), villous atrophy in celiac disease (110), chronic obstructive pulmonary disease (111), infections (69), graft injuries (65, 70, 112), cardiovascular diseases (35), Alzheimer disease (113), and polycystic ovary syndrome (114). These associations highlight the broad impact of Sb9 in various inflammatory and immune-related conditions. Furthermore, recent research has also pinpointed Sb9 as a new biomarker and potential target for various cancers (18). These findings collectively underscore the multifaceted roles of Sb9 across various health domains (**Figure 2**).

### 3.1 Cancer

Recent studies have elucidated a strong correlation between Sb9 expression and cancer progression, highlighting its potential role in tumorigenesis. Dysregulation of Sb9 has been found in high grade prostate tumors (102) and acute myeloid leukemia (115). Overexpression of Sb9 has been detected in a variety of cancers, including low grade prostate cancer (102), cervical cancer (58), gastric cancer (49), melanoma (101), nasopharyngeal carcinoma (116), glioma (63), hepatocellular carcinoma (117), non-small cell lung carcinoma (118), breast cancer (119), acute lymphoblastic leukemia (120), Hodgkin lymphomas and non-Hodgkin lymphomas (95, 121). Additionally, Sb9 is found consistently in high grade prostatic intraepithelial neoplasia and atrophic lesions (102).

As mentioned previously, the expression levels of Sb9 in tumors have been explored as a potential biomarker and prognostic indicator in cancer. For instance, in B-cell non-Hodgkin lymphoma, high Sb9 expression is associated with a higher grade of malignancy (95). Sb9 is also identified as the top risk gene for the association with oral squamous cell carcinoma, based on patient databases (122). Interestingly, Sb9 is not typically expressed in normal tissues, but expression can be acquired in oropharyngeal squamous cell carcinoma (123) and CNS-primitive neuroectodermal tumors (124). Likewise, there is significantly increased Sb9 expression in lung cancer tissues compared to non-cancer tissues, with decreased Sb9 expression in airway-derived CD8^+^ T cells from the cancer group compared to healthy controls (125). Also, Sb9 expression in primary lung cancer cells is significantly correlated with the stage of non-small cell lung cancer (125). However, non-significant differences in Sb9 expression are found between colorectal healthy and tumorous tissues or across different stages (126). The non-significant trend in Sb9 expression is also observed in tumorous tissue and regional lymph node metastases (126). Instead, increased expression of Sb9 in healthy tissue is significantly linked with longer overall survival (OS) in colorectal cancer patients, including those with distant metastases (126). High expression of Sb9 in tumor is negatively associated with prognosis and can be used to predict clinical outcome of ALK-negative anaplastic large cell lymphoma patients (127), hepatocellular carcinoma patients (117) and melanoma patients due to its negative correlation with CD8^+^ T cells (71, 94). Conversely, in nasal NK/T-cell lymphoma, Sb9 expression by tumor cells is associated with significantly better 5-year OS and 5-year event-free survival (EFS). Absence of Sb9 and low apoptotic index are related to poor outcomes (128).

The expression of Sb9 is widely acknowledged as a critical mechanism in enabling cancer cells to evade immune surveillance. Sb9 has been implicated in the modulation of immunosuppressive environments within the prostate cancer milieu (52) and has exhibited notable upregulation in bortezomib-resistant recurrent and relapsed multiple myeloma, as well as in resistant metastatic melanoma cells (106, 129). These findings highlight its promise as a therapeutic target aimed at overcoming resistance to immunotherapy treatments.

### 3.2 Infections

In renal transplants, Sb9 expression in the kidney is increased during infection with cytomegalovirus, Epstein-Barr virus or BK virus, in comparison to stable grafts (69). Similarly, up-regulation of Sb9 is also observed in lymphocytes and monocytes following infection by Epstein-Barr virus, but not in response to bacterial infection (91). Not confined to immune system, serum levels of Sb9 also rise during primary cytomegalovirus infection after transplantation, with a more pronounced increase observed in symptomatic patients than in those who are asymptomatic (130).

The role of Sb9 during infections can be complex and vary depending on the pathogen involved, indicating that its effects on the host’s immune response can be either beneficial or harmful. For example, Sb9 increases in cytotoxic lymphocytes during Ectromelia virus (Mousepox) infection, which is important to protect the host from the infection (78). On the other hand, the elimination of SPI-6 in neutrophils triggers the release of neutrophil elastase, which aids in combating Pseudomonas aeruginosa infections (93). Additionally, mycobacterium tuberculosis (MTB) can stimulate the expression of Sb9 in human mononuclear phagocytes (92, 131). The presence of Sb9 in monocytes is associated with the replication of intracellular MTB, and its inhibition can reduce the replication of MTB within cells, indicating that Sb9 protects these primary targets of infection from apoptosis, thereby enhancing the intracellular survival of the pathogen (92, 131). Mononuclear phagocytes from HIV-infected patients maintain increased activity of intracellular Sb9, which supports higher intracellular replication of MTB (131).

### 3.3 Allogeneic transplantation

It has been reported that the expression of Sb9 could regulate Treg-mediated immunosuppression in allogeneic transplantation model (32, 83). The functionality of Sb9 within host intestinal epithelial cells can be amplified by IFN-γ originating from donor T cells. This amplification serves as a protective mechanism against donor T cells, presenting a potential strategy for attenuating graft-versus-host disease (132). Conversely, a deficiency in host Sb9 exacerbates graft-versus-host disease, as evidenced by a significant increase in lethality, as well as elevated clinical and histopathological scores (132). In acute renal allograft rejection, the presence of infiltrating cytotoxic T cells within the graft initiates apoptosis of renal tubular epithelial cells. The upregulation of Sb9 within renal allografts serves as a crucial endogenous mechanism, reducing the susceptibility of renal tubular epithelial cells to cytotoxic lymphocyte attacks. Consequently, this process plays a pivotal role in preserving tubular integrity and function (69, 70). The upregulation of Sb9 expression in tubular epithelial cells is induced by proinflammatory cytokines secreted from infiltrated cytotoxic cells (65, 70). This enhanced Sb9 expression provides protection against various forms of inflammatory kidney injury and facilitates long-term allograft survival. (65, 70). In murine studies, Sb9 deficiency is observed to enhance early tubular injury and expedite the rejection of kidney allografts, confirming the pivotal role of Sb9 in inhibiting GzmB-mediated injury to renal tubular cells and promoting renal allograft survival (70). Moreover, levels of Sb9 mRNA in urinary cells vary according to the severity of acute rejection, thereby serving as a diagnostic marker. The Sb9 levels in allograft tubular epithelial cells are higher in patients without rejection compared to those experiencing rejection, accompanied by a lower frequency of apoptosis protein caspase-3 (65). The levels of Sb9 not only predict the histological grade of renal allografts but also anticipate the functional outcomes following acute rejection (65, 112).

Elevated Sb9 DNA methylation, along with reduced Sb9 expression in circulating T cells, has emerged as a novel risk factor for the onset of both initial and subsequent post-transplant cutaneous squamous cell carcinoma (cSCC), a significant complication following organ transplantation (133). Targeting the DNA methylation of Sb9 holds promise as a therapeutic approach for managing cSCC in transplant recipients (133).

### 3.4 Cardiovascular disease

GzmB is suggested to play a role in inflammation associated with atherosclerotic coronary artery diseases. Consequently, regulating GzmB through Sb9 could represent a novel therapeutic strategy for preventing cardiovascular diseases, such as atherosclerosis, plaque rupture, and ventricular remodeling after acute myocardial infarction (AMI) (134). In the progression of atherosclerosis, GzmB is prevalently found in unstable atherosclerotic plaques. It is believed to contribute to plaque instability by inducing vascular smooth muscle cells apoptosis and by degrading plaque extracellular matrix (68). In addition, atherosclerotic patients exhibit significantly higher levels of activated caspase-3 and GzmB as well as reduced levels of Sb9 in both peripheral leucocytes and vascular smooth muscle cells compared to control (68, 135). In contrast, the unaffected arterial wall contains abundant and homogeneously distribution of Sb9 (35). Therefore, GzmB and Sb9 could be effective modulators in the development and progression of atherosclerosis (135).

Conversely, retarding the apoptosis of vascular smooth muscle cells by Sb9 is a key process in pathogenesis of abdominal aortic aneurysms (AAA) (136). Hypo-methylated Sb9 gene is positively correlated with AAA patients, along with its elevated mRNA expression (136, 137).

### 3.5 HIV

HIV-infected cells with elevated levels of Sb9 can effectively evade detection and destruction by cytotoxic CD8^+^ T cells, thereby bypassing the immune system’s natural defense mechanisms against infected cells (85). Monocytes isolated from HIV-infected patients exhibit higher expression levels of Sb9 mRNA and protein compared to those from healthy individuals (131). This elevated intracellular Sb9 activity in the monocytes is associated with an increased vulnerability to infections by other pathogens (131).

## 4 Serpinb9 and therapy

Given the protective role of Sb9 in regulating cell apoptosis, the modulation of homeostasis between Sb9 and GzmB emerges as a potential therapeutic strategy for various conditions, including cancers (30), HIV, autoimmune disorders (36, 84), renal transplantation (70), and hepatitis (107).

### 4.1 Therapeutic targeting of Serpinb9 in cancer

Sb9 serves a dual role in cancer progression: it protects the body against cancer development by maintaining proper immune function, while also promoting cancer progression by assisting malignant cells in evading immune detection and destruction. Given its role in cancer, Sb9 is being studied as a potential therapeutic target, either on its own or in combination with other therapy. Modulating Sb9 could potentially enhance the sensitivity of cancer cells to immune-mediated destruction. Hence, it has become a subject of interest for researchers looking to understand and combat cancer through various therapeutic strategies.

Sb9 is identified as the top immune-resistance gene in a study of somatic gene alterations correlating with immune cytolytic activity in a TCGA cohort of lung cancer (119). Notably, it stands out as the most upregulated gene in immune checkpoint non-responders compared to responders across various cancer types, indicating its potential utility as a predictive biomarker for immune checkpoint blockade (ICB) response and a prognostic marker (50, 71, 72, 101). Sb9 expression is elevated not only in patients who are refractory to ICB therapy but also in those with post- versus pre-treatment cancers (101). High Sb9 expression in melanoma correlates with poor response to ICB (101), leading to lower survival rates following anti-immune checkpoint inhibitor therapy (50, 71). Overexpression of Sb9 reverses the potency of redirected target lysis by AMG-110, a well-characterized EpCAM/CD3-bispecific BiTE^®^ antibody construct currently undergoing clinical trials in late-stage cancer patients with various carcinomas (138). Therefore, pharmacological intervention targeting Sb9 may enhance the efficacy of immunotherapy (71, 129, 138). The resistance to CTLA4 checkpoint inhibition mediated by Sb9 justifies the therapeutic benefit of co-targeting Sb9 and CTLA4 (72).

### 4.2 Serpinb9 in T cell-mediated immunotherapies

Immune profile studies demonstrate that high Sb9 levels are closely correlated with significant T cell dysfunction (50, 72), promoting resistance to T cell-mediated killing (50). Combining Sb9 inhibition with tumor-associated antigen-directed T-cell therapy could yield superior efficacy (119). The low expression of Sb9 sensitizes cells to CD20 CAR T cell-mediated killing, indicating that Sb9 plays a crucial role in controlling tumor cell killing by CAR T cells. Combining Sb9 inhibition with CAR T treatment could enhance therapeutic efficacy (139). DNA methylation of Sb9 in circulating T cells represents a persistent risk factor for cutaneous squamous cell carcinoma (cSCC) development, which could be a future target for editing using the CRISPR/Cas9 system. Although this novel technology is far from clinical application, it holds promise for the future (133).

### 4.3 Serpinb9 in dendritic cell function and cancer vaccination strategies

Modifying the expression of Sb9 in dendritic cells (DCs) may offer a novel approach for cancer treatment. Sb9 can prolong the lifespan of DCs by delaying their apoptosis. In one study, genetic modification of dendritic cells to overexpress Sb9 enhanced their ability to prime OVA antigen-specific CTLs in semi-allogeneic recipient mice, presenting a potential cancer prevention method (140). Another research demonstrates that co-administration of Sb9 with human papillomavirus type 16 E7 antigen enhances CD8^+^ T cell-mediated E7-specific immune responses and antitumor effects more effectively than either strategy alone. These findings suggest that a vaccination strategy combining Sb9 to prolong DC lifespan could enhance vaccine potency and hold promise for future clinical applications (141). Moreover, a novel defined CD56^dim^ IFNα-induced, monocyte-derived DCs exhibit high expression of GzmB, perforin, and Sb9, which contribute to direct tumoricidal activity against K562 cells. Dissecting DCs composed of heterogeneous populations into functional subpopulations defined by molecular markers should enhance our understanding of DC biology and improve the clinical outcomes of DC vaccines (88). KO of Sb9 in mesenchymal stem cells (MSCs) results in diminished activity of downregulating cytotoxic T lymphocyte (CTL)-mediated lysis, suggesting that Sb9 in MSCs could be a potential target due to its immunosuppressive properties (30).

### 4.4 Serpinb9 in other diseases and conditions

Sb9 may play a multifaceted role in HIV treatment. The expression of Sb9 drives the resistance of HIV-1 RNA^+^ cells to CD8^+^ T cell killing, making it a potential target for HIV therapy (85). A novel two-dimensional nanosheet has been developed to enhance Sb9 expression in murine splenocytes. This enhances HIV vaccine-triggered immune responses *in vivo* by enhancing either antigen presentation or T cell-mediated apoptosis of HIV-infected cells (142).

Sb9 may exert a protective effect in certain somatic cells. For instance, it can protect tubular epithelial cells from apoptosis, thereby increasing the survival rates of renal allograft patients (65, 70). Additionally, the acute induction of GzmB contributes to fulminant hepatitis, a rapidly developing and lethal syndrome (143). Transfection of Sb9 in hepatocytes confers resistance to killing by cytotoxic T cells and hepatotoxicity, suggesting that delivery of Sb9 protein or transgene to liver might be a new strategy for prevention or treatment of GzmB-induced hepatitis (54, 107).

### 4.5 Therapeutic approaches and challenges in Serpinb9 targeted therapy

The advancement in our understanding of Sb9-GzmB axis in various physiological and pathophysiological processes has led to several therapeutic approaches for disease treatment, especially in cancer treatment. Development of Sb9 small molecule inhibitors represents an attractive approach due to its simplicity in clinical applications. Compound 3034, a small molecule inhibitor of Sb9, has recently been identified from an *in vitro* screening, which effectively restrains the growth of melanoma, breast cancer, kidney tumors, and lung cancer *in vivo* (30). However, further refinement of the structure is needed to improve the specificity and pharmacokinetic (PK) profile. SiRNA-based therapeutics represents another attractive approach to inhibiting Sb9 activity. SiRNAs have the advantage of high selectivity and may have less concern of off-target effects compared to small molecule drugs. However, *in vivo* delivery of siRNA, especially tissue-specific delivery remains a challenge despite recent progress in siRNA delivery to liver. Another interesting strategy is the use of GzmB variants that are resistant to Sb9-mediated inhibition. However, further *in vivo* studies with these candidates are necessary to evaluate their activity, as well as potential immunogenicity and nonspecific toxicity (22). All of these approaches remain at the preclinical stage. It is important to emphasize that the development and utilization of Sb9-based therapies would require extensive research and clinical trials to ensure their efficacy and safety.

## 5 Nanotechnology-based therapy through targeting Serpinb9

What is clear from the information outlined thus far is that Sb9-GZMB axis could be regarded as a promising target for different indications, notably in cancer therapy. However, a significant challenge lies in the lack of small molecules effective against Sb9 that also possess desirable pharmacokinetic profiles. To address this, quite a few nanoparticle systems have been developed to enhance the delivery of therapeutic agents targeting Sb9. These nanoparticles are particularly beneficial in targeting cancer cells while minimizing damage to healthy tissues (144–147). This selective targeting reduces potential side effects and toxicity, thereby improving both the bioavailability and antitumor efficacy (148, 149). One of the key advantages of nanoparticle-based drug delivery is the ability to engineer systems that release multiple drugs simultaneously into tumors, at a controlled rate or in response to specific triggers like pH, glutathione or reactive oxygen species (150). This controlled release mechanism ensures the maintenance of optimal drug ratios and therapeutic concentrations in the target tissue over an extended period (151). In summary, nano drug delivery systems offer a way to improve the effectiveness, safety, and patient compliance of various therapeutics, paving the way for more efficient and targeted approaches to healthcare.

One study investigated the application of protocatechuic acid (PCA), a natural phenolic acid known for its inhibitory effects on Sb9. Despite demonstrating excellent antitumor efficacy, PCA’s short half-life limits its widespread use against cancers. To address this limitation, Li and colleagues integrated PCA with isoguanosine (isoG), a natural product with strong self-assembling properties, to create a novel multifunctional supramolecular hydrogel. This PCA-based hydrogel serves as a multifunctional system with significant anticancer effects and an ability to remodel the tumor microenvironment (TME). This offers the potential for localized administration, seamlessly integrating targeted therapy and chemotherapy into a unified approach for treating oral squamous cell carcinoma (OSCC) (152). In another study, Huang et al. combined the reported Sb9 inhibitor 3034 with the immunogenic cell death (ICD)-inducing chemotherapeutic drug doxorubicin and encapsulated them into tumor-targeting peptide (Arg-Gly-Asp, RGD) decorated liposomes for breast cancer chemo-immunotherapy. This nanomedicine effectively accumulates at the tumor site, releasing doxorubicin to directly kill tumor cells and activate the host immune response through ICD induction. Additionally, the locally released 3034 relieves the inhibition of Sb9 on GzmB, facilitating the degradation of matrix components, enhancing immune cell infiltration, and improving nanomedicine penetration, ultimately leading to enhanced therapeutic outcomes (145).

Prodrug-based nanocarriers combined with photothermal therapy (PTT) represents an effective strategy for achieving a synergistic antitumor effect. Compound 3034 is conjugated to a second near-infrared (NIR-II) semiconducting polymer backbone via a glutathione (GSH)-cleavable linker. Once in the tumor, 3034 can be specifically liberated from the backbone to inhibit Sb9, thereby reactivating the activity of GzmB to enhance cancer immunotherapy. Moreover, the polymer induces photothermal therapy for direct tumor ablation, immune cell death (ICD), and the elicitation of an immune response under NIR-II photoirradiation (153). Furthermore, this well-controlled combination photothermal immunotherapy (PTI) can be synergized with other regimens to improve cancer treatment (105). A photothermal-sensitive liposome fused with M1 macrophage exosomes (M1 Exo) is constructed to efficiently transport protein GzmB and siRNA of Sb9 to the cells. This hybrid nanosystem exhibits pronounced efficacy in suppressing tumor growth in both B16 subcutaneous and lung metastasis models, offering a new strategy of multi-combinational tumor therapy (105).

As discussed before, Sb9 is a double-edged sword with different functions in different cell types especially in most tumor cells and CTLs, achieving cell-type selective delivery is crucial to minimize adverse effects on beneficial immune cells. We have recently developed a novel siRNA delivery system that targets tumor cells specifically, with minimal impact on other healthy immune cells. Furthermore, in a preliminary study, we demonstrate that the combination of siSb9 with gemcitabine (siSb9/GEM) exhibits a better synergistic effect than the combination of gemcitabine with 3034 (unpublished results). This enhanced synergy may be attributed to the siSb9/GEM combination fostering a more favorable tumor immune microenvironment (TIME), which shall bode well with combination with ICB in the future.

## 6 Summary and future direction

Numerous studies on Sb9 have been conducted, particularly in murine models. The murine orthologue of human SERPINB9 is Serpinb9 (SPI6), and at a cellular level, it plays an identical role in inhibiting GzmB. Despite functional similarities, mouse Sb9 and human Sb9 do differ in the *in vitro* kinetics of interaction with GzmB. Human Sb9 is a direct, highly efficient inhibitor of human GzmB with an SI of 10 and a K_asso_ above 10^6^ M/s, mainly due to a P1 glutamic acid, which is cleaved only by proteases with a preference for acidic residues (39). In contrast, mouse Sb9 has a nonacidic P1 residue (Cys), with a K_asso_ outside the defined physiological range with mGzmB (K_asso_=5.6 ± 1.4 × 10^4^ M/s and SI=5.8 ± 0.5) (39, 40, 154). These data suggest that while mouse Sb9 can inhibit mGzmB-mediated death, it does not directly interact with mGzmB. Recent reports have indicated that mGzmB is much less cytotoxic than hGzmB and lacks the ability to cleave the key pro-apoptotic substrate Bid due to differences in its catalytic cleft topology (39). These differences suggest that human and mouse Sb9 likely control cell apoptosis through different mechanisms, which can be explained in three non-mutually exclusive ways: other mouse Sb9 homologs are involved in cell death; this process in mice requires some cofactors; mouse Sb9 is also required to control other rodent-specific GzmB paralogues to prevent death (39, 40). Indeed, compared to humans, larger mouse Sb9 homologues are located on chromosome 13, likely reflecting the need to regulate a larger proteinase repertoire arising from differing evolutionary pressures on the reproductive and immune systems. In addition to Sb9, other orthologues of human Sb9 in mice have been characterized, including Serpinb9b/9c/9d/9e/9f/9g (155), but limited information is available regarding the structure and function of other Sb9 homologues except for Serpinb9b. Serpinb9b, which inhibits cell apoptosis mediated by granzyme M, cooperates with Sb9 in protection from effector cell-mediated cytotoxicity (156, 157). Deficiency of Serpinb9b confers leukemia resistance in SPF mice, which is controlled by activation of another kinase, receptor-interacting serine/threonine-protein kinase 2 (RIPK2) (158). These discrepancies must be considered when conducting research on Sb9 in murine models. Further studies are needed to gain a comprehensive understanding of the Sb9 homologues in mice. Moreover, besides GzmB, limited information has been revealed regarding the endogenous inhibitors of Sb9 or the pathways regulated by Sb9. Multi-omics technology is not only a powerful tool for understanding the pathways involving Sb9 but also for facilitating the development of precision medicine for optimal patient treatment (159). Furthermore, advancements in nanotechnology will contribute to improving treatment by regulating Sb9 and its associated upstream or downstream pathways.

## Author contributions

HH: Conceptualization, Investigation, Project administration, Validation, Writing – original draft, Writing – review & editing. YM: Writing – review & editing. SL: Conceptualization, Funding acquisition, Project administration, Resources, Supervision, Writing – review & editing.
